# BACE1 controls synaptic function through modulating release of synaptic vesicles

**DOI:** 10.1038/s41380-021-01166-2

**Published:** 2021-06-22

**Authors:** Brati Das, Neeraj Singh, Annie Y. Yao, John Zhou, Wanxia He, Xiangyou Hu, Riqiang Yan

**Affiliations:** Department of Neuroscience, UConn Health, Farmington, CT USA

**Keywords:** Neuroscience, Physiology

## Abstract

BACE1 initiates production of β-amyloid peptides (Aβ), which is associated with cognitive dysfunction in Alzheimer’s disease (AD) due to abnormal oligomerization and aggregation. While BACE1 inhibitors show strong reduction in Aβ deposition, they fail to improve cognitive function in patients, largely due to its role in synaptic function. We show that BACE1 is required for optimal release of synaptic vesicles. BACE1 deficiency or inhibition decreases synaptic vesicle docking in the synaptic active zones. Consistently, BACE1-null mice or mice treated with clinically tested BACE1 inhibitors Verubecestat and Lanabecestat exhibit severe reduction in hippocampal LTP and learning behaviors. To counterbalance this synaptic deficit, we discovered that BACE1-null mice treated with positive allosteric modulators (PAMs) of metabotropic glutamate receptor 1 (mGluR1), whose levels were reduced in BACE1-null mice and significantly improved long-term potentiation and cognitive behaviors. Similarly, mice treated with mGluR1 PAM showed significantly mitigated synaptic deficits caused by BACE1 inhibitors. Together, our data suggest that a therapy combining BACE1 inhibitors for reducing amyloid deposition and an mGluR1 PAM for counteracting BACE1-mediated synaptic deficits appears to be an effective approach for treating AD patients.

## Introduction

Typical clinical symptoms of Alzheimer’s disease (AD) are gradual loss of memory and cognitive ability [1–4]. The abnormal elevation of β-amyloid peptides (Aβ) in AD patients’ brains is recognized as one of the earliest changes [5–7]. Oligomeric Aβ, generated from amyloid precursor protein (APP) through sequential cleavages by β- and γ-secretase, is thought to induce a cascade of pathological events that include the formation of amyloid plaques, neurofibrillary tangles, and neurodegeneration [8]. BACE1, β-site amyloid precursor protein cleaving enzyme 1, was discovered to be the sole β-secretase that initiates the production of Aβ [9–13], and BACE1 inhibition or deletion reduces amyloid deposition in brains of AD patients and animal models [14, 15]. While potent BACE1 inhibitors significantly decrease Aβ generation and amyloid deposition in the brain, patients’ cognitive function could not be improved according to clinical ratings of dementia among prodromal AD patients [16, 17], resulting in the early termination of clinical trials.

In fact, BACE1-null mice exhibit impaired synaptic transmission and plasticity, manifested by reduced long-term potentiation (LTP) in both Schaffer collateral (SC)-CA1 synapses and in mossy fiber-CA3 synapses [18, 19]. When BACE1 is ablated in adult conditional knockout mice, despite the reversal of preexisting amyloid plaques, LTP reduction is also noted [20, 21]. This LTP reduction is also seen in the non-AD background, in line with the observation that BACE1 is required for hippocampal axonal organization [22]. Consistent with this, mice treated with BACE1 inhibitors such as SCH1682496 or LY2811376 also exhibited impaired hippocampal LTP and reduced spine formation in layer V pyramidal neurons [23]. Therefore, global and dramatic inhibition of BACE1 over a long period appears to compromise the benefit of Aβ reduction due to mechanistic side effects associated with synaptic impairment.

To understand the potential cause of synaptic impairment in BACE1-null mice, we explored molecular targets that are altered in BACE1-null mice. We discovered that synaptic vesicles were not properly docked on the synaptic active zone, and significantly fewer synaptic vesicles were in the readily releasable pool (RRP) in BACE1-null mice or mice treated with BACE1 inhibitors such as Verubecestat (MK-8931) and Lanabecestat (AZD3293). We found a clear reduction of synaptic proteins such as complexin, VAMP2, synaptophysin, and metabotropic glutamate receptor 1 (mGluR1) in BACE1-null mouse brains. Intriguingly, BACE1-null mice that were treated with a specific mGluR1-positive allosteric modulator (PAM), i.e., a previously tested compound Ro0711401 [24], reversed reduction of LTP and improved learning and memory. While two clinically tested BACE1 inhibitors Verubecestat and Lanabecestat indeed caused severe reduction in LTP in mice, this reduction can also be mitigated by mGluR1 PAM treatment. Our results suggest that BACE1 inhibitors used in conjunction with mGluR1-PAMs will be a more effective treatment of Alzheimer’s patients than BACE1 inhibitors alone.

## Results

### BACE1 deficiency reduces mGluR1 expression

**Fig. 1:**
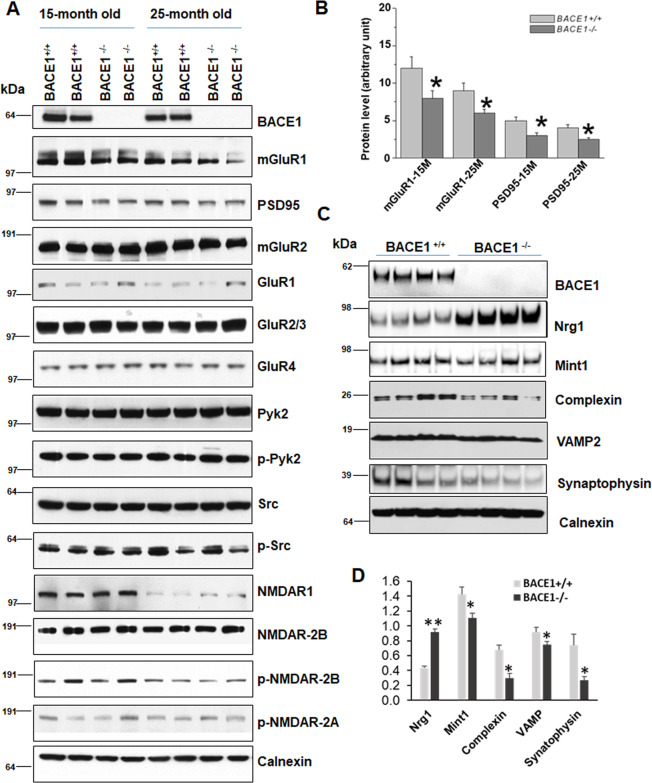
BACE1 deficiency causes reductions in synaptic proteins. **A** Hippocampal synaptosomes prepared from 15- and 25-month-old mice were examined on the blot using antibodies as indicated. While levels of most synaptic proteins were not significantly differed between wild type (WT) and BACE1-null samples, mGluR1 and PSD-95 were clearly reduced in BACE1-null samples. **B** Bar graphs showing a significant change in levels of mGluR1 and PSD-95 in aging mice (*N* = 3 independent experiments, *P < 0.05; Student’s *t* test). **C** Brain lysates from 12-month-old WT and BACE1-null mice were analyzed by antibodies as specified on the western blot. Reduction of complexin, VAMP, and synaptophysin was noted, while BACE1 substrate neuregulin-1 (Nrg1) was elevated due to abolished cleavage. **D** Bar graphs showed statistical significance (*N* = 4, **P < 0.01, *P < 0.05, Student’s *t* test).

To determine synaptic protein molecules that may contribute to the observed reduction in LTP upon BACE1 deficiency or inhibition, we isolated synaptosomes from both wild-type (WT) (BACE1^+/+^ mice with a C57/BL6 background) and BACE1-null mouse brains at different age groups to discern potential changes in synaptic proteins. Commercially available antibodies to synaptic proteins were used for western analyses. We noted a reduction of protein levels in mGluR1 and postsynaptic PSD-95 in both 15-month-old and 25-month-old hippocampi (Fig. 1A), and this reduction was statistically significant when normalized to loading controls (Fig. 1B, *N* = 3 experiments, two pairs each, *P < 0.05, Student’s *t* test). A slight decrease of mGluR5 was seen in 25-month-old but less obvious in 15-month-old brain samples (data not shown). No significant changes in protein levels of mGluR2, GluR1, GluR2/3, or GluR4 were observed. It is noted that the expressions of both PSD-95 and mGluR1 were age-dependent, and their lower levels are correlated with reduced synaptic function in aging mice [25, 26]. NMDAR1 was also lower in 25-month-old mouse brains compared to 15-month-old mouse brains, but there was no statistical significance between WT and BACE1-null mouse brains.

In hippocampal and cortical lysates from 12-month-old mice, reduction of presynaptic proteins such as complexin, VAMP2, Mint1, and synaptophysin were also noted in BACE1-null mice (Fig. 1C). Significantly increased levels of full length neuregulin-1 (Nrg1) were due to abolished cleavage of Nrg1 by BACE1 at the ectodomain [27]. It appears that BACE1 deficiency reduces levels of several pre- and postsynaptic proteins important for synaptic functions.

### BACE1 inhibitors cause synaptic impairment in mice

**Fig. 2:**
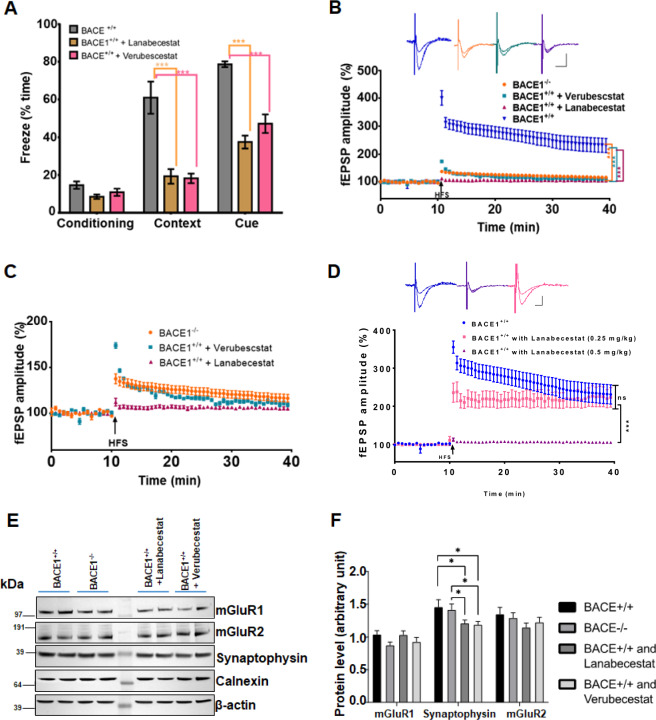
BACE1 inhibitors markedly reduce learning behaviors and long-term potentiation. **A** Fear conditioning tests were conducted in 6-month-old WT (BACE1^+/+^) mice treated with Lanabecestat (AZD3293) at 0.5 mg/kg or Verubecestat at 3 mg/kg. A significant reduction in freezing time in both contextual and cue phases in BACE1-inhibited mice (*N* = 10 in each group, ***P < 0.01, Student’s *t* test). **B** LTP was recorded on horizontal hippocampal slices from these treated mice. At 30 min post stimulation, the potentiation was 105.6 ± 0.6137% from slices treated with Lanabecestat, 113.1 ± 1.353% from slices treated with Verubecestat, lower than WT controls (BACE1^+/+^, 245.9 ± 0.9864%; *N* = 7–8, ***P < 0.001, ns meaning no significance, Student’s *t* test). The typical traces are shown in correlated colors. **C** In comparison to BACE1-null mice, WT results were removed so that fEPSP was in a narrowed range. LTP reduction by Lanabecestat or Verubecestat treatment was worse than BACE1 deficiency. **D** Lower dose of Lanabecestat (0.25 mg/kg) showed significantly less reduction on LTP compared to the higher dose (0.5 mg/kg), suggesting a dose dependent reduction in LTP by this compound. *N* = 7–8, ***P < 0.001, and ns meaning no significance, Student’s *t* test. **E** Total hippocampal lysates from mice treated with BACE1 inhibitors were examined by western analysis with the indicated antibodies. **F** Bar graphs showing a significant reduction in levels of synaptophysion in BACE1-null or inhibitors treated mice (*N* = 3 independent experiments; *P < 0.05; Student’s *t* test). Reduction of mGluR1 in BACE1-null mice was visible, but not significant (*P* = 0.65).

As aforementioned, BACE1 deletion causes reduction in LTP. The brain-penetrable BACE1 inhibitors Verubecestat (MK-8931) and Lanabecestat (AZD3293) are potent in reducing Aβ generation [28, 29]. To determine whether Verubecestat and Lanabecestat would affect learning behaviors and synaptic plasticity in mice, we treated WT (BACE^+/+^) mice with these two compounds over a period of about 4 months followed by the fear conditioning test and LTP assays for comparisons. WT mice were treated with either Verubecestat (3 mg/kg) or Lanabecestat (0.5 mg/kg) for 4 months beginning at the age of about 2 months. Standard contextual conditional fear tests were first performed after the treatment. Mice treated with either Verubecestat (3 mg/kg) or Lanabecestat (0.5 mg/kg) showed no significant differences in percentage of freezing time from mock-treated mice during the first day preconditioning test (Fig. 2A). However, percentage of context-dependent freezing time on day 2 was significantly decreased in both BACE1 inhibitors treated mice (Fig. 2A, *N* = 12–15 mice in each group; ***P < 0.001, ANOVA with Tukey’s post hoc test). Moreover, cue-dependent freezing time on day 3 also showed significant reduction in mice treated with BACE1 inhibitors. These results indicate that BACE1 inhibitors impair associative cognitive behaviors in mice.

After this test, mice with daily administration of BACE1 inhibitors were used for LTP measurements. LTP at SC-CA1 synapses in mice treated with Lanabecestat was strongly suppressed (purple trace in Fig. 2B, *N* = 8, ***P < 0.001). This reduction in LTP appeared to be more severe than that seen in the same age of BACE1-null mice (Fig. 2B, orange trace), suggesting the possible existence of an off-target effect. Mice treated with Verubecestat also exhibited significantly reduced LTP (green trace in Fig. 2B*, N* = 8, ***P < 0.001), although the detrimental effect was smaller than Lanabecestat, but still worse than that seen in BACE1-null mice (replotted in Fig. 2C for clarity). Impairment was dose dependent as lowering the dose by a half (0.25 mg/kg of Lanabecestat) showed significantly weaker LTP reduction (Fig. 2D).

In line with LTP reduction, we also found significantly reduced levels of synaptophysin on the western blots in mice treated with these two BACE1 inhibitors (Fig. 2E, F, *P < 0.05). While mGluR1 levels were visibly reduced, but not statistically significant (*P* = 0.186, WT vs BACE1-null) when total brain lysates were analyzed. It is clear that BACE1 chemical inhibition causes more severe impairment in synaptic functions, and reduction of synaptophysin is more consistent with BACE1 deficiency.

### Potentiation of mGluR1 activity ameliorates synaptic impairment in BACE1-null mice

**Fig. 3:**
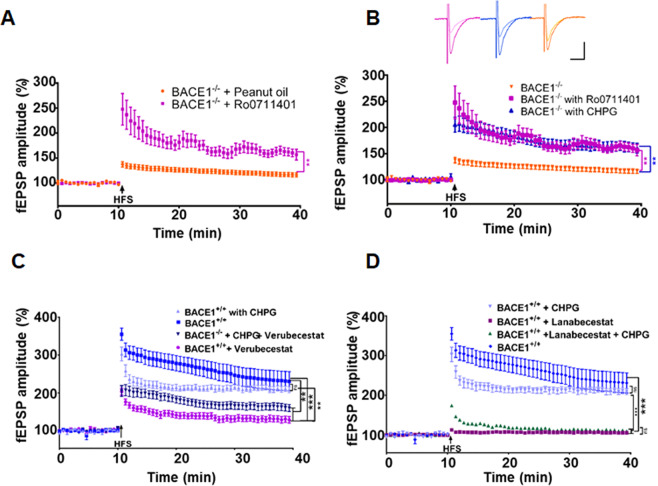
mGluR1-positive allosteric modulators LTP in BACE1-null or inhibited mice. **A** LTP was recorded on horizontal hippocampal slices from about 6-month-old BACE1-null (KO) mice treated with mGluR1-PAM Ro0711401 at 10 mg/kg, injected 1 h before sacrificing and harvesting acute brain slices. **B** BACE1-null (BACE1^−/−^) slices were treated with CHPG at 125 nM before high-frequency stimulation during LTP recording, which elicited a significantly improved LTP response when compared to mock-treated BACE1-null brain slices (*N* = 8 for BACE1^−/−^, and *N* = 11 for BACE1^−/−^ + Ro0711401 and *N* = 10 for BACE1^−/−^ + CHPG; **P < 0.01, Student’s *t* test). CHPG treatment markedly improved LTP in mice treated with Verubecestat at 3 mg/kg (**C**), but less in mice treated with Lanabecestat at 0.5 mg/kg (**D**). (*N* = 8–10; **P < 0.01; ***P < 0.001; Student’s *t* test).

Since mGluR1 at excitatory synapses modulates the strength of synaptic transmission by inducing LTP in various brain regions [30], we postulated that a reduction of mGluR1 might contribute to decreased LTP in BACE1-null mice. To test this, we treated 4–6-month-old BACE1-null mice with an mGluR1-PAM Ro0711401 (provided by Roche), which is brain penetrable and has previously been tested in animals [31–33]. By following the published protocol [33], we administered 10 mg/kg of Ro0711401 in BACE1-null mice via intraperitoneal (IP) injection 1 h before the animal was sacrificed for LTP recordings. Remarkably, LTP measured from acutely sectioned brain slices was significantly enhanced compared to BACE1-null mice treated only with vehicle (peanut oil) (Fig. 3A; *N* = 5, **P < 0.001, Student’s *t* test), suggesting that acute Ro0711401 treatment rapidly reverses LTP reduction at SC-CA1 synapses.

Both mGluR1 and mGluR5 belong to the group I metabotropic glutamate receptors [34]. The compound (RS)-2-chloro-5-hydroxyphenylglycine (CHPG; TOCRIS) was initially developed to be a specific PAM of mGluR5 for modulating synaptic transmission and neuronal excitability, but later shown to be equally potent to both mGluR1 and mGluR5 [35]. We then tested this compound in our LTP measurement. Since CHPG is nonbrain penetrable, we pretreated BACE1-null slices with 125 nM CHPG for 30 min followed by LTP measurement. A significantly improved LTP response was also noted (blue traces in Fig. 3B). Hence, acutely treated mGluR1-PAMs appear to have a potentiation effect on LTP in the BACE1-null mouse brain.

To test whether the reduced LTP due to BACE1 inhibition would also be affected by mGluR1-positive modulators, we treated about 6-month-old WT mice with Verubecestat (3 mg/kg) or Lanabecestat (0.5 mg/kg) for 60 days by oral gavage. For LTP measurements, we bath-treated brain slices with CHPG as described above. Compared to mock treatment, a markedly improved LTP response was noted when CHPG was incubated with brain slices from animals treated with Verubecestat (Fig. 3C, blue trace, ***P < 0.001). Mice treated with Lanabecestat exhibited stronger reduction in LTP when compared with Verubecestat in our tests. Notably, CHPG treatment had only slightly improved LTP responses in mice treated with Lanabecestat (Fig. 3D, green trace). Hence, mGluR1-positive allosteric modulation will mitigate the LTP reduction by Verubecestat treatment, but this effect is much less pronounced for Lanabecestat treatment in mice.

### Improved psychiatric and memory behaviors in BACE1-null mice treated with an mGluR1-PAM

While in vitro or acute treatment with mGluR1-PAMs mitigates synaptic impairment in BACE1-null mice, it is not understood whether there is a cognitive benefit in animals. To this end, we conducted chronic in vivo treatment of BACE1-null mice with the brain-penetrable PAM Ro0711401 (Roche). Beginning at the age of about 12 months, BACE1-null mice were either given Ro0711401 by IP injection at a dose of 10 mg/kg or vehicle (peanut oil) every 12 h during the testing period of 45 days. Both learning behaviors and LTP were examined sequentially in treated mice.

**Fig. 4:**
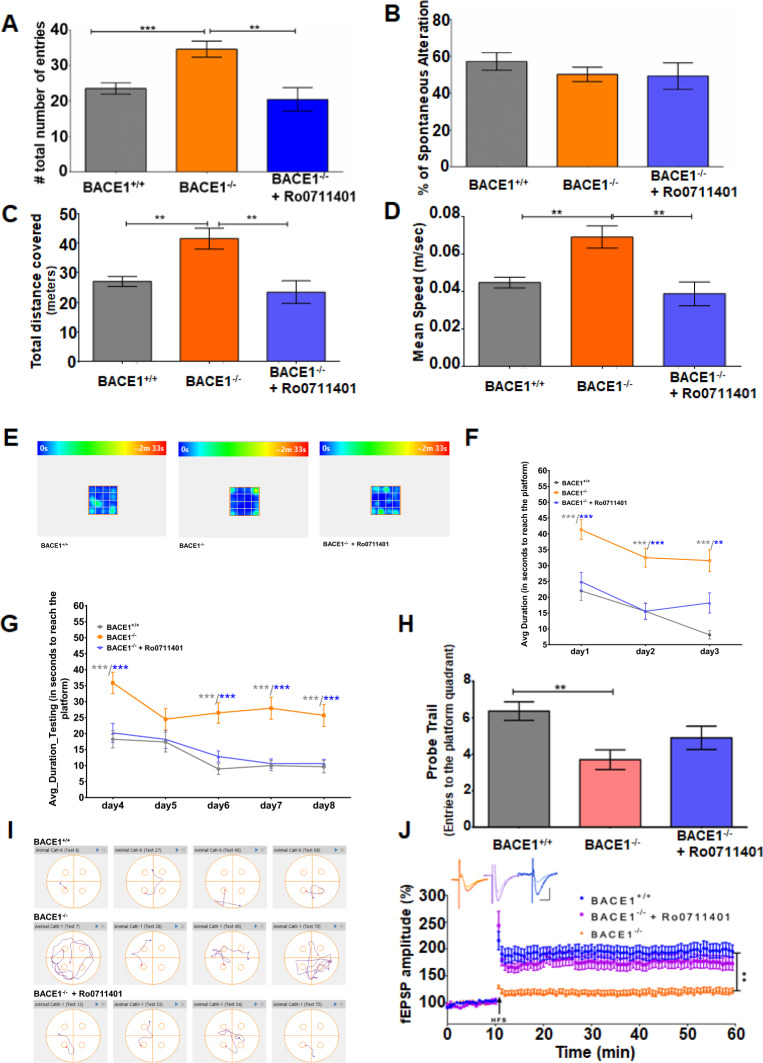
mGluR1-positive allosteric modulator ameliorates defects in learning and memory in BACE1-null mice. **A**, **B** BACE1-null mice treated with Ro0711401 were first tested on the Y-maze task in comparison to mock-treated controls. Total numbers of arm entry were shown in **A**, and changes in percentage of spontaneous alternation were not obvious among three groups shown in **B**. BACE1-null mice exhibited high numbers of entry. This increase was reverted by Ro0711401 treatment. **C–E** BACE1-null mice with or without Ro0711401 were tested in an open field. Significant reversions in the total distance covered (**C**) and average speed (**D**) were observed in BACE1-null mice treated with Ro0711401 compared to mock treatment. **E** Heat maps showing that mice treated with Ro0711401 explored both the corners and the center of the open-field arena similar to WT, while BACE1-null mice spent more time in the corners. The anxiety seen in BACE1-null mice was mitigated by PAM treatment. **F–J** Mice were tested in the Morris water maze. In the habitation (**F**) and training (**G**), BACE1-null mice showed defects in learning, taking much longer time compared to WT and BACE1-null treated with Ro0711401. **H** On day 9 in the Morris water maze test, the number of entries made to the platform quadrant looking for the hidden platform were visibly increased when BACE1-null mice were treated with Ro0711401, and this is more obvious in the representative track plots (**I**). *N* = 12 each in WT and BACE1^−/−^ group and 10 in BACE1^−/−^ with Ro0711401 treatment; **P < 0.01; ***P < 0.01; Student’s *t* test. Values are expressed as mean ± SEM. **J** LTP was recorded on horizontal hippocampal slices from 16-month-old mice as described above after treatment with Ro0711401 for ~50 days. The LTP in the Ro0711401 treatment group was comparable to WT in this age group and both were significantly higher than BACE1-null mice at 16 months (*N* = 7 for BACE1^−/−^, and *N* = 10 for BACE1^−/−^ + Ro0711401, *N* = 8 for WT; **P < 0.01, unpaired Student’s *t* test).

We first conducted the Y-maze spontaneous alternation task test to evaluate behaviors of short term spatial working memory. BACE1-null mice clearly showed increased total numbers of arm entries, reflecting more active in exploratory behaviors (Fig. 4A), consistent with a prior observation [36]. Interestingly, BACE1-null mice treated with Ro0711401 for about 45 days reduced performance in total arm entrance. Spontaneous alternations in the Y-maze was slightly reduced in BACE1-null mice (Fig. 4B), and the significantly reduced spontaneous alternation will be seen if larger numbers are included in the test [36]. It appeared that Ro0711401 treatment would not alter spontaneous alternations in BACE1-null mice.

We then conducted the open-field test to further assess the impact of Ro0711401 on exploratory behaviors associated with BACE1 deficiency. BACE1-null mice significantly traveled longer distances and moved with a faster speed than their WT counterparts (Fig. 4C, D*; N* = 12, **P < 0.001, Student’s *t* test). After treatment with Ro0711401, the total distance covered by the treated mice in the open-field arena was lowered along with their average speed (Fig. 4C, D; *N* = 12, **P < 0.001, Student’s *t* test). In addition, we noted that BACE1-null mice were mostly running along the corners and spent much less time in the central space in the open-field arena when compared to WT mice (Fig. 4E; *N* = 12, *P < 0.05, Student’s *t* test), likely attributed to schizophrenia-like behaviors as suggested [37]. BACE1-null mice were visibly shown to spend more time spent in the center after the Ro0711401 treatment. This observation suggests that application of Ro0711401 can modulate this anxiety behavior in BACE1-null mice.

In order to evaluate effects on long-term spatial memory, we also performed water maze experiments. In Morris water maze experiments, spatial learning and memory in mice rely on distal cues to navigate from start locations around the perimeter of an open swimming arena to locate a submerged escape platform. Spatial learning is assessed across repeated trials and reference memory is determined by preference for the quadrant of the maze when the platform is absent [38]. WT and BACE1-null mice with or without Ro071141 treatment were habituated in the water maze for 3 days over four separate trials on each day. BACE1-null mice took a longer time to learn to reach the platform (Fig. 4F; 41.32 ± 3.131 s on day 1 and 31.56 ± 3.432 s on day 3) when compared with WT (21.99 ± 2.986 s on day 1 and 8.098 ± 1.318 s on day 3). BACE1-null mice treated with Ro0711401 showed clear improvement (24.88 ± 2.935 s and 18.20 ± 3.203 s in BACE1-null + Ro0711401 mice, *N* = 12, ***P < 0.001). Over the next 5 days, the platform was submerged in water and the primary cue (a flag attached to the platform) was removed. During this training phase, we again observed that the average duration to reach the platform for BACE1-null mice was significantly longer than the WT and the BACE1-null mice treated with Ro0711401 over all trial days (Fig. 4G; *N* = 12, ***P < 0.001, Student’s *t* test).

On the 9th day, the platform was removed and mice were left in the water maze for 60 s to test probe capability. Ro0711401 treatment improved the number of entries made to the platform quadrant compared to BACE1-null mice (Fig. 4H, 6.364 ± 0.5094 in WT vs 3.700 ± 0.5385 in BACE1-null vs 4.900 ± 0.6403 in Ro0711401-treated group, **P < 0.001, *N* = 12 each Student’s *t* test). We also analyzed swimming behaviors, which were manifested by track plots. The plot visibly showed improved learning behaviors in mice treated with Ro0711401 in reaching the platform (Fig. 4I). Moreover, the average distance that animals commuted to reach the platform during the habituation and the training phases was analyzed. The average distance covered over the trials by BACE1-null mice was longest (3.872 ± 0.7645 m); WT mice, in contrast, traveled only 1.205 ± 0.2805 m, while BACE1-null mice with Ro0711401 treatment traveled 1.909 ± 0.3878 m (Fig. 4I, *N* = 12; ***P < 0.001 between WT and BACE1^−/−^ and *P < 0.05 between BACE1^−/−^ and BACE1^−/−^ + Ro0711401; Student’s *t* test), showing improvement in learning to reach the platform at the end of training phase. Collectively, these data show that Ro0711401 treatment in BACE1-null mice improved behaviors in open field and water maze tests.

After the behavioral tests, mice were evaluated for their synaptic plasticity measured by LTP, induced by high-frequency stimulation (HFS) in hippocampal slices. Consistent with the ameliorated learning and exploratory behaviors, LTP was significantly elevated to the level close to that of WT mice at the age of 14–15 months old (Fig. 4J). Synaptic impairment due to BACE1 deficiency can be significantly reversed when Ro0711401, a potent mGluR1-PAM, is given to mice for about 2 months.

### Impaired synaptic plasticity by BACE1 inhibitors is mitigated by mGluR PAMs

To determine whether mGluR1 PAMs would also alleviate the detrimental effect on LTP in mice treated with BACE1 inhibitors, we administered 12-month-old BACE1^+/+^ animals with either Verubecestat at 3 mg/kg or Lanabecestat at 0.5 mg/kg by oral gavage daily along with another potent mGluR1 PAM Ro67-7476 at 4 mg/kg, i.p. (Tocris Bioscience cat # 4346), which has similar potency to Ro0711401 [39]. After mice were treated with either the BACE1 inhibitor alone or in combination with PAM Ro67-7476 for ~50 days, we conducted the behavioral tests as discussed above to assess the impact on cognitive behaviors associated with BACE1 inhibition.

**Fig. 5:**
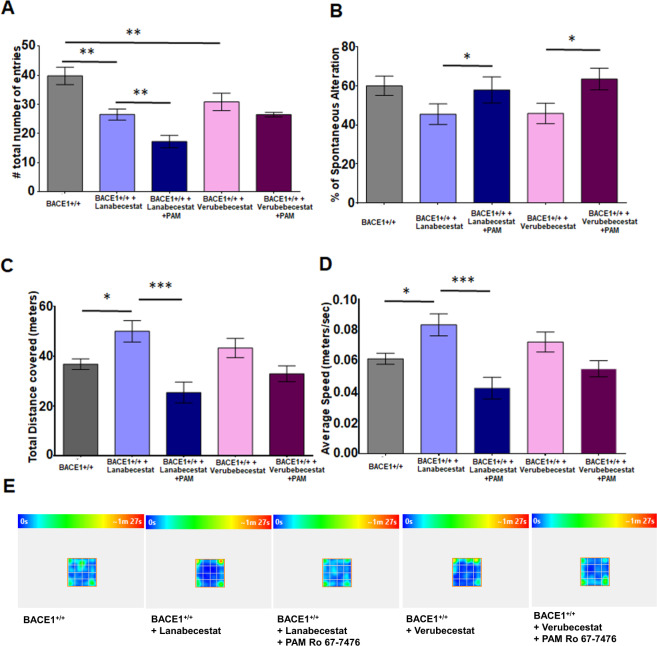
mGluR1-positive allosteric modulator reverses impaired cognitive behaviors by the BACE1 inhibition. **A**, **B** Administration of mGluR1 PAM Ro67-7476 improved the number of % spontaneous alteration in Y-maze task when given in mice treated with BACE1 inhibitors Lanabecestat or Verubecestat. Unlikely seen in BACE1-null mice, the number of total entries was markedly reduced in mice treated with these two inhibitors. **C**–**E** These mice were also tested on the open filed tasks. A marked reduction in the total distance covered and average speed in the open field in the combined treatment group of mgluR1 PAMs and BACE1 inhibitors vs only BACE1 inhibitors. The heat maps in **E** showed improved anxiety behaviors in BACE1-inhibited mice (*N* = 10 in BACE1^+/+^, 10 in BACE1^+/+^ + Lanabecestat, 10 in BACE1^+/+^ + Verubecestat, 9 in BACE1^+/+^ + Lanabecestat + Ro67-7476, and 10 in BACE1^+/+^ + Verubecestat + Ro67-7476: **P < 0.01; ***P < 0.01; Student’s *t* test).

On Y-maze test, unlike BACE-null mice (Fig. 4A), BACE1^+/+^ mice treated with either Verubecestat or Lanabecestat had reduction in total entry, and a further reduction was noted when Ro67-7476 was included in the treatment (Fig. 5A, **P < 0.05 between WT and Verubecestat group, *N* = 9, Student’s *t* test). This reduction was more obvious in mice treated with Lanabecestat either alone or together with Ro67-7476 (Fig. 5A, **P < 0.01 and ***P < 0.001, *N* = 9, Student’s *t* test). Mice treated with either Verubecestat or Lanabecestat showed small reduction in spontaneous alteration behaviors, but no statistical significance (Fig. 5B). Mice in Verubecestat + Ro67-7476 group showed improvement in spontaneous alternation when compared to mice treated with Verubecestat alone (Fig. 5B, *P < 0.05, *N* = 9, Student’s *t* test). Similarly, mice in the Lanabecestat + Ro67-7476 group improved spontaneous alternation over mice in with only Lanabecestat (Fig. 5B, *P < 0.05, *N* = 9, Student’s *t* test). Our results indicated that BACE1 inhibition has differential effect on Y-maze behaviors compared to BACE1-null mice.

BACE1-null mice exhibited anxiety behaviors by traveling more on the open field as shown in Fig. 4C, and mice treated with either Verubecestat or Lanabecestat also traveled greater distances (Fig. 5C) and faster (Fig. 5D). WT mice treated with the cocktail of BACE1 inhibitors and mGluR1 PAM significantly reduced their traveled distances (Fig. 5C), and with slower speeds (Fig. 5D, *N* = 9, Student’s *t* test). Among these two compounds, we noted that the speed was reduced more in the Lanabecestat + Ro67-7476 group than in the Verubecestat + Ro67-7476 group. Mice treated with either Lanabecestat or Verubecestat stayed more in the corner of the open-field area than mock-treated controls (Fig. 5E). Adding mGluR1 PAM in the treatment improved open-field results slightly in the Verubecestat group, while not much improvement was observed in the Lanabecestat group (Fig. 5E).

Together, our data show that impaired cognitive behaviors associated with BACE1 inhibition can be partially ameliorated by mGluR1 PAM. Along with LTP assays shown in Fig. 3C, D, we demonstrate that the combo treatment in mice not only mitigates LTP reduction but also partially improves learning behaviors.

### BACE1 deficiency decreases releases of synaptic vesicles

**Fig. 6:**
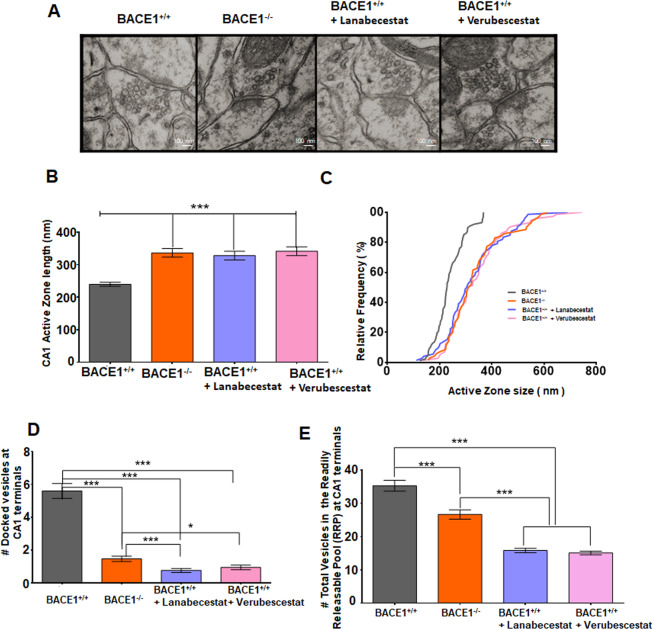
BACE1 deficiency or inhibition decreases release of synaptic vesicles in the active zones. **A** Representative EM images show different active zones and synaptic vesicles in the presynaptic compartment of WT control (BACE1^+/+^), in BACE1 deficient mice BACE1^−/−^, or BACE1^+/+^ mice with Lanabecestat or Verubecestat. **B**, **C** Active zone lengths and their cumulative frequency histogram in above conditions were quantified. **D** Number of docked vesicles (within 5 nm from presynaptic membrane) per active zone. **E** Synaptic vesicle distribution up to 200 nm distance from presynaptic membrane (constituting RRP) of the active zone (*N* = 3 animals in each group, 87 randomly selected active zones or 60 synapses were analyzed; ***P < 0.001, unpaired Student’s *t* test).

In our biochemical assays, we showed that both BACE1 deficiency and inhibition by compounds commonly cause reduction in synaptophysin, this result was in line with our morphological examination, which showed impairment in the active zone ultrastructure (Fig. 6A). Specifically, a significant increase in average active zone length, measured on transmission electron microscopy (TEM) images, was seen in BACE1-null mice compared to WT controls (Fig. 6B; 239.7 ± 6.293 nm in BACE1^+/+^ vs 336.8 ± 13.17 nm in BACE1^−/−^, *N* = 87, ***P < 0.001, Student’s *t* test). As compared to the WT, the active zone length was also increased to 341.3 ± 13.49 nm in mice treated Verubecestat and 327.8 ± 13.40 nm in BACE1^+/+^ treated with Lanabecestat (*N* = 75, **P < 0.01, ***P < 0.001; unpaired Student’s *t* test). BACE1 deficiency or inhibition caused a consistent rightward shift in the curve measuring the relative frequency of active zone lengths (Fig. 6C).

We also measured synaptic vesicle docking, defined as those synaptic vesicles within 5 nm of the membrane [40], as well as distribution of synaptic vesicles. A marked decrease in numbers of synaptic vesicles docked on the active zone was (Fig. 6D) observed in BACE1-null conditions when compared with the WT. Quantification showed that an average of 5.59 ± 0.45 synaptic vesicles in WT vs 1.48 ± 0.16 synaptic vesicles in BACE1^−/−^ (quantified from *N* = 60 TEM images, 5 synapses in each image, ***P < 0.001). Compared to WT controls, a significant decrease in docked vesicles was also seen in brains from mice treated with these two BACE1 inhibitors (Fig. 6D, 0.77 ± 0.12 in the Lanabecestat group and 0.95 ± 0.14 in the Verubecestat group, *N* = 60, ***P < 0.001).

In addition, we also measured total vesicles within 200 nm from the active zone (Fig. 6E). The total average number of vesicles within 200-nm from presynaptic membrane or the RRP was 35.31 ± 1.64 in BACE1^+/+^ vs 26.69 ± 1.41 BACE1^−/−^ (*N* = 60, ***P < 0.001). BACE1 inhibitors caused even more reduction of RRP (15.83 ± 0.64 in the Lanabecestat group and 15.08 ± 0.57 in the Verubecestat group, *N* = 60, ***P < 0.001). The more severe reduction of the RRP in mice treated with BACE1 inhibitors is correlated with more severe reduction in LTP (Fig. 3C).

**Fig. 7:**
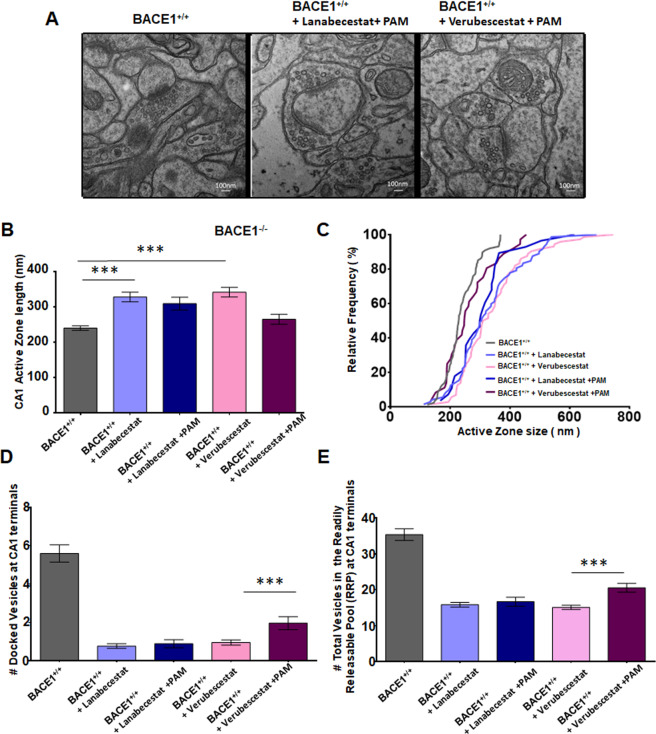
mGluR1 PAM treatment impacts active zone length. **A** Representative EM images show synaptic structures in WT mice treated with mGluR1 PAM Ro677467 together with Lanabecestat or Verubecestat. **B**, **C** Active zone lengths and their cumulative frequency histogram were quantified and compared among four treated groups and WT like above and show a relatively increase in size in the animals treated with BACE1 inhibitors alone or in combination with PAMs. **D** Number of docked vesicles (within 5 nm from presynaptic membrane) per active zone is also significantly decreased. **E** Synaptic vesicle distribution up to 200 nm distance from presynaptic membrane of active zone is also impacted in the groups with BACE1 inhibitor treatments (*N* = 60, ***P < 0.001, Student’s *t* test).

We further examined whether mGluR1 PAM treatment would affect synaptic vesicle by examining mice treated with combo (BACE1 inhibitor + mGluR1 PAM Ro67-7476). Our results showed that mGluR1 PAM treatment had marginal effect on the synaptic vesicle release. The synaptic ultrastructure was not altered, but synaptic vesicles were still less in contact with the active zone in Lanabecestat + PAM (Ro67-7476) and Verubecestat + PAM groups when compared to WT controls (Fig. 7A). Quantification of the length of active zone showed only slightly reduction in the Lanabecestat + PAM group (309.0 ± 18.12 nm vs 327.8 ± 13.40 nm), but no statistical significant (Fig. 7B; *N* = 60). Interestingly, PAM treatment had relatively more reduction on the active zone length in the Verubecestat + PAM group (264.7 ± 14.17 nm in Fig. 7B) compared to the Verubecestat group (341.3 ± 13.49 nm; *N* = 60, ***P < 0.001, Student’s *t* test). There is a relative rightward shift of the active zone size when plotted as relative frequency histogram (Fig. 7C).

Similarly, the docking of synaptic vesicles in the active zone was also only affected in the Verubecestat + PAM group (Fig. 7D; 0.95 ± 0.14 in the Verubecestat group vs 2.0 ± 0. 34 in the Verubecestat + PAM group, and 5.60 ± 0.45 in WT mice, *N* = 60, ***P < 0.001, Student’s *t* test). No significance was noted in the Lanabecestat group vs the Lanabecestat + PAM group (0.77 ± 0.12 vs 0.89 ± 0.21) group, *N* = 60, ***P < 0.001, Student’s *t* test). The trend in the total number of synaptic vesicles in the RRP (SVs with 200 nm from the presynaptic membrane) was also consistent (Fig. 7E; 15.83 ± 0.64 in the Lanabecestat group, 16.67 ± 1.26 in the Lanabecestat + PAM group, 15.08 ± 0.57 in the Verubecestat and 20.52 ± 1.22 in the Verubecestat + PAM group, *N* = 60, ***P < 0.001, Student’s *t* test). Together, PAM treatment had a marginal improvement on synaptic release in mice treated with Verubecestat, but almost no effect on the Lanabecestat treatment.

## Discussion

BACE1 is a prime target for AD therapy, as it is elevated in the brains of patients with AD, accumulated in the swollen axons and its cleavage of APP is the rate-limiting step in Aβ production [41, 42]. However, clinical trials of BACE1 inhibitors in treating AD patients have not been successful because of their failures to improve cognitive functions, despite reducing Aβ plaque load [16, 17]. Therefore, it is critical to find solutions that will take advantage of this plaque reduction while overcoming the unwanted side effects associated with worsening cognitive functions/scores. We demonstrate that mGluR1-PAM in combination with a safe dose of BACE1 inhibitor is a promising combination therapy for treating AD patients.

In AD brains, two well-studied hippocampal circuits (the mossy fiber-CA3 synapse and SC-CA1 synaptic pathways) are severely impaired, likely attributable to oligomeric Aβ toxicity [43, 44]. Inhibition of BACE1 should improve Aβ-mediated synaptic impairments. However, chronic deletion of neuronal BACE1 in adult mice causes reduced LTP [20, 21], in line with significantly reduced synaptic release demonstrated in this study (Fig. 6). The disorganization of the infrapyramidal bundle comprised of axons of dentate gyrus granule cells may also partially explain this hippocampal synaptic impairment [22]. Hence, BACE1 is clearly required for the control of synaptic strength. Strong inhibition of BACE1 in humans or animals will complicate the potential cognitive benefit resulting from reduced Aβ-mediated toxicity.

One important on-target synaptic impairment by BACE1 chemical inhibition is the reduced number of synaptic vesicles within the RRP (Fig. 6D, E). In synapses, a presynaptic terminal is densely packed with large number of vesicles, only small fractions of which are available for immediate release. The RRP size and the probability of release of each vesicle within that RRP, on a whole, determine synaptic strength of that terminal [45]. Only the vesicles, which are already primed and docked at the presynaptic membrane, are ready for undergoing exocytosis and releasing their content for neural transmission upon synaptic stimulation [46, 47]. BACE1 is present in presynaptic vesicles [48], and BACE1 inhibition or deficiency clearly impacts the active zone nanoarchitecture and reduce synaptic vesicle docked at synaptic zones. Perhaps, this structural defect is related to observed reduction of synaptic fusion proteins such as complexin and VAMP2 in BACE1-null mice (Fig. 1C, D). Small but significant reduction of synaptophysin was also consistently seen in mice with BACE1 deficiency or deficiency. Consequently, this reduction would cause a reduced LTP.

**Fig. 8:**
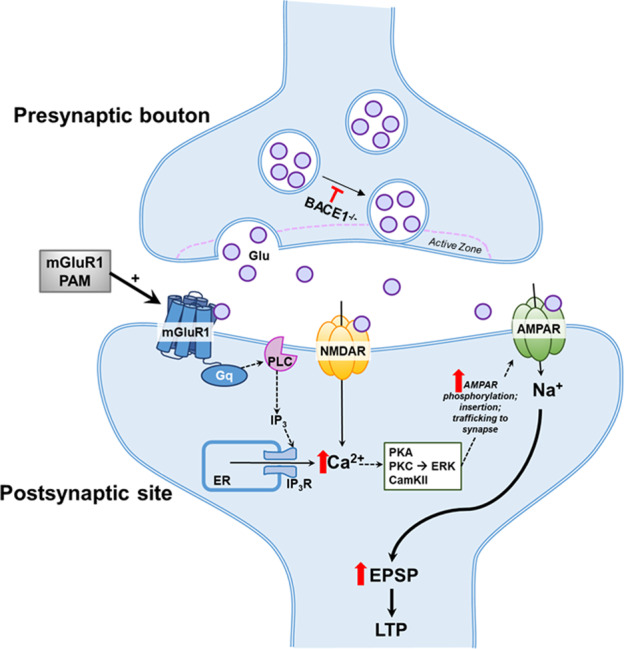
Schematic illustration of synaptic transmission by mGluR1 PAM treatment. BACE1 inhibition or deficiency causes reduction in distributed of synaptic vesicles to the active zone. This causes less number of glutamate (Glu) released from these exocytosis, and weaker activation of NMDA and AMPA receptors on the postsynaptic membranes. mGluR1 PAM counteracts such a reduction by inducing more calcium (Ca^2+^) release through G-coupled receptor G_q_-PLC-IPs pathway.

In this study, we aim to explore how to improve synaptic strength if BACE1 is inhibited. BACE1 has many characterized substrates such as Nrg1, Nrg3, and SEZ6; all these are known to play important roles in synaptic transmission and plasticity [49–51]. In our western blot analyses, we were intrigued by a reduction of mGluR1 in BACE1-null brains (Fig. 1), as Nrg1 can regulate the expression of synaptic mGluR1 [52]. Since BACE1 deficiency or inhibition will reduce the cleavage of Nrg1 by decreasing its signaling between Nrg1 and its cognate receptor ErbB4, this reduction can lead to reduced expression of mGluR1 as demonstrated in this study [53]. Reduction of mGluR1 has attracted our particular attentions as mGluR1 was also reduced in AD brains [54]. mGluR1 is one of eight identified mGluRs, which are seven transmembrane nonionotropic G-protein coupled receptors, and is predominantly localized on the postsynaptic membrane [34]. The binding of glutamate to mGluR1 on the excitatory postsynaptic membrane induces certain forms of LTP by increasing inflow of Ca^2+^ and potentiating and NMDAR activity as illustrated in Fig. 8. Here, we showed that, by supplementing BACE1-null mice the mGluR1-PAM such as Ro0711401, reduced LTP was markedly reversed or mitigated. The effect of mGluR1 PAM does not appear to reverse the reduced release of synaptic vesicles, but counteracts the reduced synaptic transmission because of BACE1 deficiency or inhibition.

More remarkably, we noted significantly improvement in learning and psychiatric behaviors by the mGluR1 PAM in either BACE1-null mice or mice treated with BACE1 inhibitors. Mice with BACE1 deficiency exhibit anxiety behaviors as well as impaired learning and memory, as assessed in the Y-maze (Fig. 4A), open field (Fig. 4C–E) and Morris water maze (Fig. 4F–I), and in line with prior observations [37]. By giving BACE1-null mice the PAM for 45–60 days, we showed that chronic activation of mGluR1 significantly improved anxiety behaviors as well as learning and memory in BACE1-null mice, consistent with the improvements in LTP measures, indicating the beneficial effect of PAM.

Considering the need of BACE1 for optimal synaptic function in mice, we evaluated two clinically tested compounds, Verubecestat (MK-8931) and Lanabecestat (AZD3293), in our mouse study. We found that mice treated with either Verubecestat or Lanabecestat caused even more severe reduction of LTP at SCs to CA1 synapses than BACE1 deficiency (Fig. 2D). Although both compounds potently inhibited BACE1, their impairments on LTP were differential: Lanabecestat at 0.5 mg/kg had a stronger effect on LTP reduction than Verubecestat at 3 mg/kg, and the potentiation was not stable over time. This observation suggests that chronic usage of BACE1 inhibitors at high dose might actually be detrimental in exhibiting and maintaining synaptic plasticity. In clinical trials, BACE1 inhibition was found to reduce hippocampal volume [55], a phenotype not yet confirmed in BACE1-null mice and whether this is related to the possible off-target effect remains to be determined. It should be aware that BACE1 likely process human and rodent substrates in different efficacies [56], and this difference may contribute to subtle variations in synaptic phenotypes. However, an encouraging note is that a lower dose of Lanabecestat shows significantly reduced synaptic toxicity (Fig. 2D), suggesting the potential benefit for testing a lower dose in human trails

Comparing animal behavioral tests in mice treated with BACE1 inhibitors or deficiency, we noted some consistency and differences. Mice treated either BACE1 inhibitor consistently displayed impairment in cognitive functions including learning behaviors (Fig. 2A) and anxiety (Fig. 5) in two different age groups. In our Y-maze and open-field tests, we found that mice treated with BACE1 inhibitors behaved differently: less active on the Y-maze, manifested by reduced total arm entry, compared to BACE1-null mice (Fig. 5A, B compared to Fig. 4A). Although differed from BACE1-null mice, mice in both inhibitor groups appeared to be consistent (Fig. 5A, B). On the open field, all these groups of mice were more timid, spent less time in the center of the open field compared to WT. Such difference may likely be attributable to potential inhibition of BACE1 homologs such as BACE2, which has different substrates [57, 58]. Although BACE1 inhibitors did not significantly decrease mGluR1 level (Fig. 2E), mGluR1 PAM mitigated aforementioned behaviors impaired in all these BACE1-inhibited or deleted groups.

In summary, our study in mice treated with these two BACE1 inhibitors showed significantly impaired synaptic plasticity and cognitive functions, consistent with the decision of terminating these BACE1 inhibitor compounds in human trials. Nevertheless, BACE1 inhibitors have been shown to be extremely effective in reducing Aβ levels and slowing the amyloid deposition in AD, long-term use of BACE1 inhibitory drugs should benefit AD patients by reducing pathological accumulation. Such a benefit should be realized as an early event. We have shown that the dose of Lanabecestat lowering to 0.25 mg/kg significantly diminished LTP reduction (Fig. 2D), and testing lower dose for realizing the benefit of BACE1 inhibitors should be explored in animals and human. Considering both BACE1 inhibitors showed similar synaptic toxicity, safe dose of BACE1 inhibitors may avoid cross inhibitions of other aspartyl proteases including BACE2. While BACE1-mediated and Aβ-mediated neural networks appear different [59], combining an mGluR1-PAM with an optimal dose of BACE1 inhibitory drugs will likely provide a complementary way to treat AD patients. Hence, future strategies to use BACE1 inhibitors that can override any mechanism-based side effects and reduce off-target side effects should be further investigated and explored.

## Material and methods

### Animals

BACE1-null mice were generated as previously described [60] and maintained in a C57BL/6 background. All animal use and procedures were performed according to the Institutional Animal Care and Use protocols at Cleveland Clinic and UConn Health Center, Farmington, and in compliance with the guidelines established by the Public Health Service Guide for the Care and Use of Laboratory Animals. Animals of either sex were used.

### Drug delivery

C57BL/6 mice of either sex (4–6 months of age; *N* = 6 per group) received vehicle or BACE1 inhibitors, Lanabecestat (AZD3293) solution at 0.25 mg/kg or 0.5 mg/kg) via oral gavage [61]. For Verubecestat, 3 mg/kg dose was used and delivered orally via gavage [28]. Treatment usually lasts 2–4 months. The mGluR1-PAM Ro0711401 at 10 mg/kg was given as IP injections 1 h before decapitation for LTP experiments [33]. For behavioral assays, Ro0711401 was delivered via IP injection at 12-h intervals for 6–8 weeks. The mGluR1/5 PAM CHPG was mixed in artificial cerebrospinal fluid (aCSF) and used during recording LTP. The drug was applied in a bath during baseline recording for at least for 30 min before HFS stimulation was applied and LTP was recorded [62]. Ro67-7476 was injected intraperitoneally at 4 mg/kg at 24 h intervals for 8 weeks before behavior or LTP assays were performed in combination with Lanabecestat or Verubecestat at the above dosages for combined drug studies.

### Western blotting and antibodies

Synaptosome isolation was performed using Thermo Scientific Syn-PER Synaptic Protein Extraction Reagent (cat # 87793) from whole brain samples. Briefly, mice were sacrificed by live decapitation, and rapidly dissected hippocampi were homogenized in Syn-Per reagent, ∼1 ml/100 mg tissue. Samples were then centrifuged at 1200 × *g* for 10 min. The resulting supernatant was then centrifuged at 15,000 × *g* for 20 min. Pellets (synaptosomes) were resuspended in buffer B (3 mM sucrose in 6 mm Tris, pH 8.0) with 1% SDS, briefly sonicated, and frozen in −80 °C. Total protein extraction was performed according to previously described procedures [63]. Brain samples were homogenized in radioimmunoprecipitation assay buffer (50 mM Tris-HCl, pH 7.4, 1% NP-40, 0.25% sodium deoxycholate, 150 mM NaCl, 1 mM EDTA, 1 mM NaF, 1 mM Na_3_VO_4_, and a protease inhibitor cocktail [Roche]) and centrifuged at 13,200 rpm for 90 min. Equal amounts of protein were resolved on a NuPAGE Bis-Tris gel (Invitrogen) and transferred onto nitrocellulose membranes (Invitrogen). Subsequently, blots were incubated with the following primary antibodies overnight at 4 °C: Bace1 (3599) [1:1000, rabbit pAb, gift from Dr Xiaoxin Yan’s lab]; mGluR1 (D5H10) [1:1000, Cell Signaling #12551]; PSD-95 (7E3) [1:1000, Cell Signaling #36233]; mGluR2 (D7D8M) [1:1000, Cell Signaling #76012]; GluR1 (C3T) [1:5000, Upstate #05-855]; Glur2/3 [1:1000, Upstate #07-598]; GluR4 [1:1000, Upstate # 06-308]; Pyk2 [1:1000, Cell Signaling #3480]; p-Pyk2 [1:1000, Cell Signaling #3291]; Src [1:1000, Cell Signaling #2123]; p-Src (D7F2Q) [1:1000, Cell Signaling # 12432]; NMDAR1 [1:250, Chemicon AB1516]; NMDAR2B (D15B3) [1:1000, Cell Signaling #4212]; p-NMDAR-2B (Ser1284) [1:1000, Cell Signaling #5355]; p-NMDAR-2A (Tyr1246) [1:1000, Cell Signaling # 4206]; Calnexin [1:3000, Sigma C4731]; actin-beta [1:10,000, Sigma A5441]; Bace1 (D10E5) [1:1000, Cell Signaling #5606]; Nrg1 [1:1000, Millipore Ab5551]; neuregulin-1 (H210) [1:1000, Santa Cruz SC-28916], Mint-1 [1:000, Synaptic Systems 144103]; Synaptophysin [1:400, Sigma S5768]; and Vamp2 (FL-118) [1:200, Santa Cruz SC-13992]. After extensive washing, blots were reacted with their corresponding HRP-conjugated secondary antibodies (1:1000 Goat Anti-Mouse, AB_2307392, Jackson ImmunoResearch; 1:2000 Goat Anti-Rabbit, AB_2313567, Jackson ImmunoResearch) and visualized using enhanced chemiluminescence (Thermo Scientific). Western blot quantification was done using ImageJ, and protein levels were normalized to the loading controls (beta-actin or calnexin). Statistics were calculated by Student’s *t* tests.

### Slice preparation

Acute brain slices were prepared as previously described [20]. Briefly, the mice were decapitated and the brains were immersed in low-calcium aCSF containing (in mM): 125 NaCl, 2.5 KCl, 10 glucose, 25 NaHCO_3_, 1.25 NaH_2_PO_4_, 0.4 L-ascorbic acid, 3 myo-inositol, 2 Na-pyruvate, 3 MgCl_2_, and 0.1 CaCl_2_ continuously bubbled with 95% O_2_ and 5% CO_2_ (pH ≈ 7.3). Animals were decapitated with a guillotine and the brain was quickly removed to an ice-cold aCSF chamber with constant bubbling of 95% O_2_ and 5% CO_2_. Acute horizontal brain slices containing the hippocampus were obtained using a Campden 7000smz-2 vibratome from mice (at different ages as mentioned above) at 350 μm thick. Slices were immediately transferred to an incubation beaker containing normal aCSF (same as the low-calcium aCSF except that 1 mM MgCl_2_ and 2 mM CaCl_2_ were used) at 30 °C, continuously bubbled with 95% O_2_ and 5% CO_2_. Slices were allowed to recover for 1 h and then were transferred to an MED-64 recording chamber with the same aCSF solution at ≈30 °C, maintained by MED ThermoConnector (Automate Scientific) with continuous aCSF perfusion at 2 ml/min.

### LTP recordings

LTP recordings on hippocampal slices were performed according to previously described procedures [64–66]. Upon obtaining horizontal hippocampal slices from the brains of mice, the prepared slices were then placed onto the center of an MED probe (MED-P515A; AutoMate Scientific) with continuous perfusion of aCSF and bubbling of 95% O_2_ and 5% CO_2_. The device has an array arranged in an 8 × 8 pattern of 64 planar microelectrodes across a hippocampal slice. Each electrode is 20 × 20 µm with an interelectrode distance of 150 µm. A MED-A64HE1S head amplifier and a MED-A64MD1 main amplifier, run by Mobius software, were used for data acquisition and analysis. SCs to CA1 synapses were typically analyzed for LTP assays. fEPSPs caused by stimulation were recorded at a 20-kHz sampling rate within the CA1 subregion of the hippocampus. Control fEPSPs were recorded for at least 10 min before the conditioning stimulation, using a response ~50% of the maximum. After a stable baseline was established, LTP was induced with three trains of 100 Hz for 1 s with an intertrain interval of 20 s. Field potential amplitudes were then measured. Data are expressed as mean ± SEM. Synaptic strength was evaluated by measuring changes in the fEPSP amplitude relative to baseline. Statistics were calculated by Student’s *t* tests.

### Y-maze

Spontaneous alternations were tested as described previously [67]. This learning task does not involve any training, reward, or punishment and allows the assessment of spatial working memory that is dependent upon the hippocampus. The symmetrical Y-maze made of acrylic consists of three arms separated by 120°. Each arm is 40 cm long, 17 cm high, 4 cm wide at the bottom, and 13 cm wide at the top. Each mouse was placed in the center of the Y-maze and was allowed to explore freely through the maze during a 5 min session. The sequence and total number of arms entered was recorded. Arm entry was considered to be completed when the hind paws of the mouse had been completely placed in the arm. Percentage of alternations is the number of triads containing entries into all three arms divided by the maximum possible alternations (the total number of arms entered − 2) × 100. Statistics were calculated by Student’s *t* tests.

### Open field

The square white open-field arena had a diameter of 100 cm and 55-cm-high sidewalls. Each subject was released in the middle of the arena and observed for 10 min. Performance in the open field was recorded by a computer-based video tracking system (Any-maze software, San Diego instruments, USA). Activity measures included distance traveled, percentage of time spent in corners vs the center of the arena, and speed of movement during active exploration. The number of entries to the central zone of the open field was also recorded. Statistics were calculated by Student’s *t* tests.

### Morris water maze

The Morris water maze test was used to assess cognitive impairment. The apparatus consisted of a white circular tank (120 cm in diameter and 40 cm deep) filled with warm water (22 °C). The water was made to appear opaque using white nontoxic paint. A transparent platform (8 cm in diameter) was located in the middle of the southwest quadrant. Mice were subjected to four consecutive trials each day over a 3‐day training period (with a flag attached to the platform) and then again for 5 days (no flag attached to platform) and the platform was submerged 0.5 cm below the water surface. Each mouse was released from four different positions around the perimeter of the tank (north, northwest, east, and southeast). In each trial, every mouse was allowed to swim until it found the platform (for a maximum of 60 s) and was subsequently left on the platform for 20 s between trials. If the platform was not found in 60 s, the mouse was guided to the platform and remained there for 20 s. The escape latency to find the hidden platform was automatically recorded using a video tracking system (Any-maze, San Diego instruments). A probe test was conducted for 30 s on the 9th day. The platform was removed; each was released from the northeast quadrant and was allowed to swim for 60 s. Memory retention was measured by quantifying the time spent in the target quadrant, the number of entries made into the southwest quadrant, and the number of crossings over the previous platform location. Statistics were calculated by Student’s *t* tests. Values are expressed as mean ± SEM.

### Electron microscopy

WT (C57/BL6J), BACE1 KO, and drug treated (BACE1 inhibitors alone or in combination with PAMs) mice were anesthetized with ketamine and xylazine cocktail (ketamine (100 mg/kg) and xylazine (10 mg/kg) of body weight, i.p.) and perfused transcardially with 0.1 M phosphate buffer (PB), pH 7.2) followed by fixative solution for 15 min containing 4% paraformaldehyde (PFA), 2.5% glutaraldehyde, in 0.1 M PB, pH 7.2. Brains were postfixed with 4% PFA in PB overnight and 100 μm coronal sections of the hippocampus were obtained on a vibratome (Campden 7000smz-2). Tissue was rinsed with 0.1 M sodium cacodylate buffer, postfixed with 1% osmium tetroxide, 1.5% potassium ferrocyanide in 0.1 M sodium cacodylate buffer, en bloc stained with aqueous 1% uranyl acetate, and dehydrated in ethanol. Following infiltration in resin tissue was embedded in Poly/Bed 812 and polymerized at 60oC for 48 h. Ultrathin sections (70 nm) were stained with 6% methanolic uranyl acetate and lead citrate and examined in a Hitachi H-7650 transmission electron microscope operating at 80 kV. Images used for quantification were taken at ×30,000 magnification with AMT camera and software.
